# A Scoping Review of Urban Planning Decision Support Tools and Processes That Account for the Health, Environment, and Economic Benefits of Trees and Greenspace

**DOI:** 10.3390/ijerph21010048

**Published:** 2023-12-28

**Authors:** Yonatal Tefera, Veronica Soebarto, Courtney Bishop, John Kandulu, Carmel Williams

**Affiliations:** 1 School of Public Health, The University of Adelaide, North Terrace, Adelaide, SA 5000, Australia; courtney.bishop@adelaide.edu.au (C.B.); carmel.williams@adelaide.edu.au (C.W.); 2 Centre for Health in All Policies Research Translation, South Australian Health and Medical Research Institute, North Terrace, Adelaide, SA 5000, Australia; 3 School of Architecture and Civil Engineering, The University of Adelaide, North Terrace, Adelaide, SA 5000, Australia; veronica.soebarto@adelaide.edu.au; 4 School of Economics and Public Policy, The University of Adelaide, Adelaide, SA 5000, Australia; john.kandulu@adelaide.edu.au

**Keywords:** urban planning, decision support tools, economics of trees and greenspace, cost–benefit analysis, health cost, environmental cost

## Abstract

Compelling evidence shows that trees and greenspaces positively impact human well-being and the environment and offer economic benefits. Nevertheless, there exists a knowledge gap regarding the extent to which this evidence is efficiently incorporated into existing urban planning decision-making processes. This scoping review identified the extent to which urban planning decision-making frameworks, models, and tools consider the health, environmental, and economic benefits of trees and greenspace. Out of 28 reviewed studies, 11 (39%) reported on frameworks, models, and tools that take into account the health, environmental, and economic dimensions of trees and greenspace. Additionally, seven studies provided comprehensive coverage of at least one of the three key dimensions. However, none of the decision support frameworks, models, or tools comprehensively integrated all three dimensions, with only two tools (7%) scoring above 50% (five or more out of nine) in terms of comprehensiveness. This review highlights the urgent need to incorporate the true economic and monetary values of the health and environmental benefits of trees and greenspace to inform urban development decision making.

## 1. Introduction

The intricate linkages between urban planning, greenspace, trees, health, and the environment are evident through the recognition that health is influenced by a wide array of factors, encompassing social, economic, political, cultural, and environmental aspects, collectively referred to as the social determinants of health [1]. Likewise, the environment is significantly shaped by external factors and decisions that extend beyond traditional environmental boundaries [2]. Urban planning, for example, can transform the environment we inhabit by providing access to trees and greenspace, which influences physical health, mental well-being, and ecosystems, thus altering the relationship between overall human health and the environment. This highlights the need for “healthy urban planning processes” that create a balance between social, environmental, and economic priorities while considering the potential implications of decisions on both human health and the environment [3].

In urban planning and design, the terms “trees” and “greenspace” are often used interchangeably and are sometimes considered together or defined inconsistently, but they have different characteristics [4,5,6]. For instance, trees are generally less fragmented and form larger patches than shrubs, while grass typically has medium fragmentation but forms the largest patches and is more interconnected than trees and shrubs [7]. In this context, the term “greenspace” is used to encompass any area of land that is covered with vegetation, including trees, shrubs, grasses, and other types of vegetation found in urban landscapes such as parks, gardens, backyards, streetscapes, forests, and even rooftops.

Evidence supporting the health and environmental benefits of urban greening has grown significantly in recent years [8,9]. Greenspace offers a range of ecosystem services, such as pollutant reduction, temperature regulation, and the control of air and sound pollution [10]. Greenspace also plays a vital role in replenishing groundwater, recycling and conserving water, averting surface runoff and flooding, preventing soil erosion, and serving as habitats while fostering biodiversity [8,11]. Furthermore, trees and greenspace contribute significantly to environmental well-being, including carbon sequestration, energy conservation, and the reduction in carbon emissions [12]. Trees provide significant erosion and flood mitigation benefits due to their intricate root systems, height, and extensive canopies that facilitate infiltration of rainwater into the ground, interception of rainwater, and storage of rainwater within branches, trunks, and leaves, thereby reducing the amount of runoff that reaches the ground and the amount of surface runoff [13,14]. These ecosystem services, in turn, translate into health benefits by encouraging physical activities such as walking and cycling; fostering social interaction; and preventing diseases that might otherwise arise [15]. Notably, numerous studies have established positive correlations between exposure to greenspace and enhanced mental well-being, encompassing improvements in attention, mood, and cognitive functioning [16,17,18,19,20]. In addition, access to greenspace has been linked to reduction in crime [21] and increased social cohesion and social capital [22]. Research has also demonstrated various physical health benefits, including reduced risk of cardiovascular diseases [23], obesity [24], heart rate, and diastolic blood pressure [18,25].

Quantifying the mental health benefits associated with trees and greenspace remains a challenge despite a growing body of research that suggests that trees and greenspace can positively impact mental health [26,27,28]. For instance, one study found that individuals residing in neighborhoods with greater tree cover exhibited lower rates of depression and anxiety [29]. Another study revealed that spending time in nature can effectively reduce stress levels and enhance mood [30]. However, quantifying the mental health benefits of trees and greenspace remains challenging due to methodological difficulties with establishing causal relationships between exposure to trees and greenspace and mental health outcomes [31]. Isolating the effects of trees and greenspace from other factors influencing mental health, such as social factors, economic factors, and personal characteristics, presents a significant challenge.

The advantages in terms of health and the environment can be transformed into economic benefits through an increase in productivity (as healthier individuals are generally more productive) and a decrease in healthcare and environmental costs [12]. Prevention of heat-related illnesses during extreme heatwaves leads to fewer ambulance calls and visits to the emergency department, resulting in lower expenses associated with treatment and care [12,32]. Similarly, the carbon sequestration potential of trees and greenspace contributes to mitigating climate change, resulting in fewer instances of wildfires and floods, thereby reducing property damage and the costs of emergency services [33]. The heat regulation function of trees and greenspace can also lead to savings in energy consumption through a reduced need for air-conditioning [34]. Additionally, trees and greenspace can make a direct economic contribution by attracting tourists and improving environmental quality, which, in turn, increases property values [35].

Despite compelling evidence supporting the positive impacts of trees and greenspace on health, the environment, and the economy, there exists a notable gap in translating this evidence into urban planning and development decision-making processes. Some studies have noted that traditionally, the decision-making landscape has not adequately considered health and environmental benefits when making urban planning decisions, with public health and environmental issues typically falling under the responsibility of ecologists and health professionals [15,36]. The majority of prior research has concentrated on formulating frameworks that link urban planning decision-making processes with the impact on regulating ecosystem services. These studies consistently pointed out that regulating ecosystem services is often overlooked in current decision-making processes [37,38]. While these investigations generally center on ecosystem services, the significance of trees and greenspaces for these services [39] prompts the extension of the same principle to encompass trees and greenspace. Consequently, trees and greenspace are frequently seen as liabilities rather than assets, relegating them to low priority in urban planning projects, particularly when resources are limited [15]. This results in a decline in both the quantity and quality of trees and greenspace over time [12], leading to urban environments that expose people to greater health risks and environmental degradation. However, there is a growing recognition of the importance of integrating health and environmental considerations into urban planning processes, programs, and projects [40].

This status quo underscores the critical need for urban planning decision-making processes that take into account the full range of benefits that greenspace provides, including the health, the environment, and the economic benefits. Yet, there is a gap in our understanding regarding the degree to which existing urban planning decision-making processes, including frameworks, models, and tools used, integrate these interconnected benefits. In this scoping review, we systematically identified and reviewed relevant studies that examine existing urban planning decision support frameworks, models, and tools, with the specific aim of evaluating the extent to which current urban planning decision-making processes incorporate health, environmental, and economic benefits of trees and greenspace.

## 2. Materials and Methods

This scoping review was conducted in accordance with the JBI methodology for scoping reviews [41]. The Preferred Reporting Items for Systematic Reviews and Meta-Analyses extension for Scoping Reviews (PRISMA-ScR) checklist was used to guide the reporting [42].

### 2.1. Review Questions

The scoping review procedure, including the search strategy as well as the screening and selection criteria, was guided by the following review questions:

- To what extent do current decision-making processes used in urban planning projects take account the health and environmental benefits of exposure to trees and greenspace?
- To what extent do these decision-making processes incorporate the economic valuation of the environmental and/or health benefits of trees and greenspace?

### 2.2. Search Strategy

A literature search was conducted in four databases: PubMed, Scopus, Public Health Database, and Web of Science. Search terms were identified under three key concepts, “green space”, “decision-making process”, and “urban planning”, which were used to construct the logic grids. Relevant subject headings and MeSH terms for each database were identified, and the Boolean operators “AND” and “OR” were used to construct search strings. The development of the search strategy and logic grids was conducted in consultation with a research librarian (Appendix A). The grey literature search was conducted using Google Advanced Search. In particular, the websites for the relevant government departments from each Australian state and territory were searched, as well as publications by international bodies such as the World Health Organization, the World Bank, and the United Nations. Search results were imported into Covidence systematic review software, Veritas Health Innovation, Melbourne, Australia, where duplicates were identified and removed.

### 2.3. Screening and Selection

Based on the review questions, the primary concept of this review was decision-making processes that address health, environmental, and/or economic benefits of trees and greenspace. Based on this, records that fulfill all the following selection criteria were considered eligible for the review: records that (1) discuss decision-making frameworks, models, or tools; (2) incorporate at least one of the health, environmental, and/or economic benefits of trees and greenspace; and (3) are within the context of urban planning. Studies that did not consider at least one element of trees and greenspace benefits, those that discuss blue spaces, and those that focus solely on the health or environmental benefits of trees and greenspace, without any relevance to urban planning and decision support processes were excluded. An abstract and title screening was conducted using the inclusion and exclusion criteria. Each record was double-screened by two independent reviewers (C.B. and Y.T.), and any discrepancies were resolved through discussion, with inputs from a third reviewer (C.W. or V.S.) when necessary. Subsequently, full texts were assessed, and those records that met the inclusion criteria progressed to the data extraction stage. The selection and screening process was documented using a PRISMA flow diagram [42].

### 2.4. Data Extraction and Synthesis

Information from the records included in the scoping review was gathered using a data extraction template developed by the reviewers (Table A7 in Appendix B). The data extraction template comprised various fields regarding the study characteristics, such as year of publication and study area. Moreover, the template includes specific information about the decision support frameworks, models, and tools. This encompassed their creation and development process, strengths, limitations, and whether they considered health, environmental, and economic aspects of trees and greenspace.

**Figure 1:**
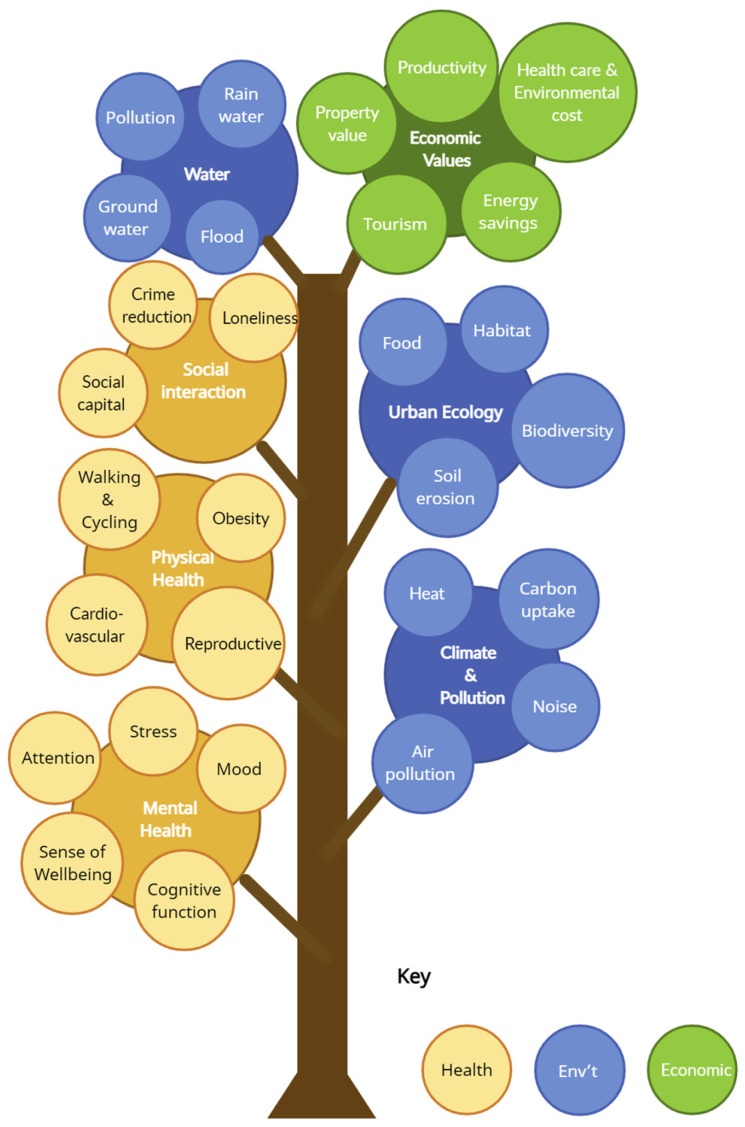
Conceptual framework used to evaluate included decision-making frameworks/models/tools.

After extracting the data, we synthesized the information and categorized the records into three primary dimensions: health, environmental, and economic, either individually or in combination. Refer to Figure 1 for a conceptual framework that serves as a guideline to assess the extent to which the identified decision support frameworks, models, and tools comprehensively incorporate any of these benefits.

The data synthesis involved scoring each literature record from 0 to 9, based on how well the reported decision-making tools and frameworks integrated health, environmental, and economic benefits of trees and greenspace with reference to the elements outlined in Figure 1 as a framework. A total score out of 9 was calculated by summing the scores across three dimensions (health, environment, economics), each rated from 0 to 3. A score of 0 in any dimension indicated no incorporation of associated benefits. For health and environmental dimensions, a score of 1 meant integration of one element (e.g., improved social capital or carbon sequestration), while a score of 2 indicated integration of two or more elements without comprehensive coverage. A score of 3 was awarded for comprehensive integration of multiple aspects of health or environmental benefits. As for the economic dimension, a score of 1 was assigned if the record addressed one or more direct economic benefits of trees and greenspace (such as tourism or property value), without considering any economic benefits linked to health or the environment. A score of 2 was assigned if it included at least one element of the economic values associated with health (e.g., avoided treatment costs of heat-related illnesses,) or environmental benefits (monetary value of air pollution removal) without factoring in any direct economic benefits. If it incorporated at least one element of the direct economic values along with at least one element pertaining to either health or environmental benefits, the study received a score of 3.

## 3. Results

### 3.1. Search Results

**Figure 2:**
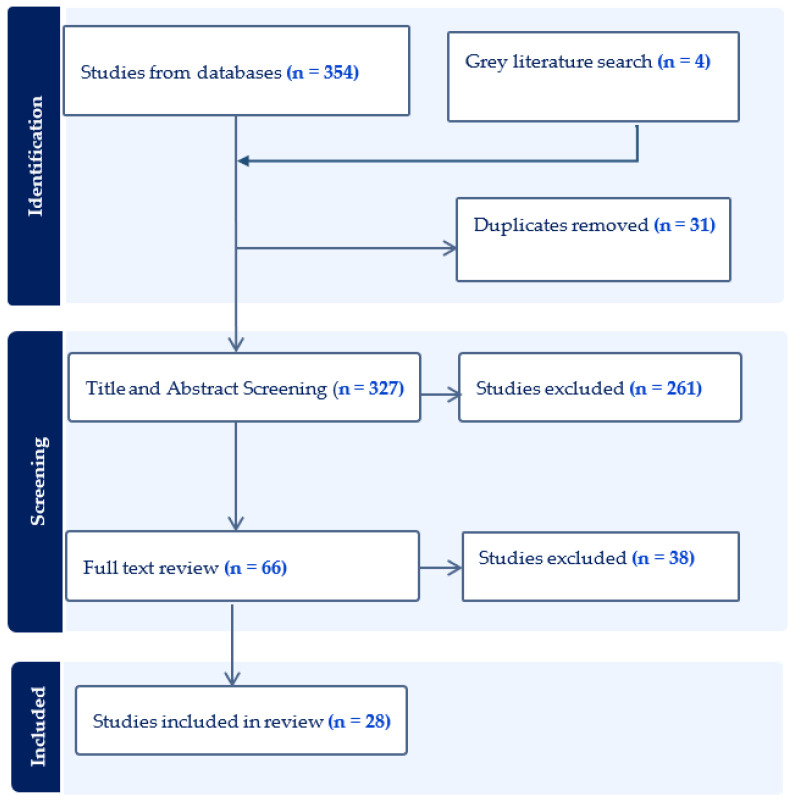
Scoping review flow diagram, adapted from PRISMA [25].

The initial database and grey literature searches retrieved a total of 358 records. After removing 31 duplicates in Covidence, 327 records went through title and abstract screening, of which 66 were retained for full text review. After full text review and screening, we ultimately included 28 records in the final review and synthesis. The remaining records were excluded for various reasons. Some did not encompass at least one aspect related to the benefits of trees and greenspace, or they focused on urban planning decision-making processes unrelated to trees or greenspace. Additionally, some solely concentrated on the health or environmental benefits of trees and greenspace without any relevance to urban planning and decision support processes. Others failed to present urban planning frameworks, models, or tools, or these frameworks and tools had already been addressed in another record included in the review. This process can be viewed in the PRISMA diagram in Figure 2.

**Figure 3:**
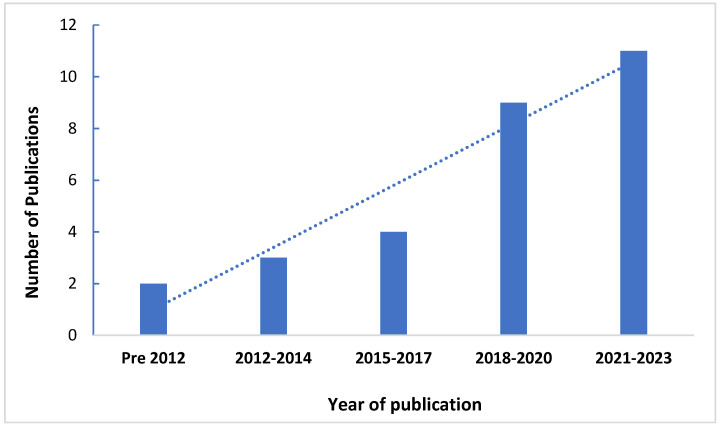
The temporal trend of publications discussing health, environmental, and economic benefits of trees and greenspace within the urban planning context.

Figure 3 shows the number of relevant publications over time. Out of the 28 studies included in this review, only 2 (7%) were published prior to 2012 [43,44]. From 2012 to 2017, the annual publication count ranged from zero to two studies, while between 2018 and 2023, this increased to three to five studies per year, except for 2020 when no studies were published. A significant portion of these studies (71%) emerged within the last six years, indicative of a growing trend in both publications and research interest regarding the integration of trees and greenspace benefits into urban planning decision-making processes. The majority of these studies were from developed nations (71%), with a smaller proportion carried out in developing nations (29%) (Appendix B).

### 3.2. Description of Tools and Frameworks

Among the 28 studies reviewed, 10 (36%) were categorized as decision-making tools, 10 (36%) were identified as frameworks, and 4 (14%) were characterized as models. Furthermore, the remaining studies included one study each that presented a matrix, a tool kit, a tool suite, and an index. Among all the studies, only 10 (36%) reported decision support processes that existed in digital formats. A range of methods were employed in the development of these decision frameworks, models, and tools, including stakeholder engagement [45,46,47,48], literature reviews [49,50,51,52], primary data collection [43,44,53], and secondary data analysis [54,55,56,57,58,59,60,61]. Some studies combined methods, such as utilizing both literature reviews and primary data [62,63], both literature reviews and secondary data [64,65,66], both stakeholder engagement and secondary data [67], and both primary and secondary data [68]. One study employed all four methods in the development of their tool [69], while another customized a preexisting framework followed by expert testing [70].

### 3.3. Integration of Greenspace Benefits into Decision-Making Processes

Table 1 presents a summary of the synthesized results and scores for the reviewed frameworks, models, and tools based on how well they incorporate health, environmental, and economic benefits of trees and greenspace. Among the 28 studies analyzed, several patterns emerged: 11 studies [43,44,45,46,48,49,50,59,65,66,69] demonstrated the incorporation of at least one element from each of health, environmental, and economic dimensions; 5 studies integrated benefits from both the environmental and economic dimensions [47,51,56,57,58]; and 5 studies [51,56,60,64,67,68] combined health and environmental dimensions simultaneously.

#### 3.3.1. Health Benefits

Nearly two-thirds (64%) of the reviewed frameworks/models/tools (18 out of 28) incorporated at least one aspect of the health benefits associated with trees and greenspace. Nevertheless, only one of these, namely, the community health framework [56], provided a comprehensive integration of multiple aspects of health benefits, albeit without considering the environmental and economic dimensions of trees and greenspace benefits. Another tool that exclusively concentrated on health benefits was the WIND tool for assessing walkability [53]. Importantly, the remaining 16 out of 18 frameworks/models/tools in this category extended their scope to incorporate at least one aspect of environmental or economic benefits alongside the health dimension.

#### 3.3.2. Environmental Benefits

The majority (26 out of 28, 93%) of examined frameworks/models/tools incorporated at least one environmental element of greenspace benefit. Among these, only six (21%) provided comprehensive coverage of multiple aspects of environmental benefits. While 21 (75%) of these extended their scope to encompass human health benefits and/or the economic values of trees and greenspace, in addition to environmental benefits, the remaining 5 incorporated benefits exclusively from the environment’s perspective. This group included Hellwig’s mathematical algorithm tool [62], a habitat suitability model [55], an urban heat island (UHI) assessment matrix [61], a performance index framework [63], and an integrated UCmap and GIS tool [60]. A common limitation among these tools was their specificity, with four out of the five tools either tailored to address UHI or focused on the suitability of greenspace to support biodiversity.

#### 3.3.3. Economic Benefits

In comparison to the health and environmental dimensions, a smaller proportion, specifically 16 out of 28 (57%) of the reviewed studies, reported frameworks/models/tools that encompassed at least one economic benefit of trees and greenspace. All of these frameworks/models/tools also considered at least one element from the health or environmental dimensions. However, only one, the I-Tree Eco tool [43], was deemed comprehensive (scoring 3 out of 3), as it integrated direct economic benefits as well as the economic values associated with the health benefits of trees and greenspace. Conversely, although another tool included multiple economic benefits in its framework, it did not quantify the monetary value of trees and greenspace benefits [46].

### 3.4. Comprehensiveness of the Tools and Frameworks

Among the 28 frameworks, models, and tools we reviewed, none comprehensively incorporated the health, environmental, and economic benefits of trees and greenspace. However, eleven of these tools and frameworks [43,44,45,46,48,49,50,59,65,66,69] did include elements from each of the three dimensions of greenspace benefits. In terms of comprehensiveness score, the overall assessment revealed that only two tools (7%) scored over 50% (5 or more out of 9). Furthermore, 13 tools (46%) scored 4 out of 9, while the remaining 13 tools (46%) scored 3 or less out of 9, as indicated in Table 1.

The two most comprehensive tools identified in this review are the I-tree [43] and the Melbourne Green Factor Tool [52]. The i-Tree, developed by the USDA Forest Service in 2006, comprises a suite of software tools and applications for evaluating the economic and environmental benefits of urban trees and greenspace. It includes tools like i-Tree Eco, i-Tree Canopy, and i-Tree Hydro, which serve urban planners, arborists, and policy makers in managing greenspace. Specifically, i-Tree Eco, the tool of primary relevance to this scoping review, leverage species, city, pollution, and weather data as input to assess greenspace structure, environmental impact, and community value. The Melbourne Green Factor Tool is a web-based application specifically developed to assess private development proposals using a green factor score calculated based on the environmental and human health benefits of greenspace incorporated in the proposal. It calculates a green factor score considering the environmental and human health benefits of greenspace integrated into the proposals. A more in-depth analysis of the strengths and limitations of these two tools is provided in the Discussion section.

## 4. Discussion

The primary aim of this scoping review was to provide an overview of the existing urban planning decision-making processes and the extent to which these incorporate health, environmental, and economic benefits associated with trees and greenspace. The results show that none of the studies identified in this review from existing publications reported urban planning decision-making processes that sufficiently and comprehensively encompass health, environmental, and economic dimensions of tree and greenspace benefits. Out of the 28 tools and frameworks examined, 11 addressed at least one element from each of the three dimensions, and 6 provided comprehensive coverage of one of the three key dimensions. However, none of them managed to comprehensively integrate multiple aspects from all three dimensions. In fact, only two tools achieved a comprehensiveness score of 50% or more, with just one of them, i-Tree Eco Tool [26], comprehensively addressing two of the three major dimensions (environmental and economic dimensions). While several tools and frameworks do integrate multiple aspects of environmental benefits, there is still a noticeable gap when it comes to incorporating health and economic benefits of trees and greenspace. Most importantly, there is a significant gap in the current urban decision-making processes in terms of incorporating economic or monetary values associated with the health and/or environmental benefits of trees and greenspace, with only 1 out of the 28 reviewed tools and frameworks partly addressing this [26]. In the sections below, we discuss the strengths and limitations of the two most comprehensive tools we identified in our review: i-Tree Tools [26] and the Melbourne Green Factor Tool [35].

The i-Tree Eco tool [26] uses input data (species, city, pollution, weather) to assess greenspace structure, environmental impact, and community value. It calculates benefits such as pollution reduction, health improvements, carbon storage/sequestration, hydrological effects, and energy savings. It also estimates the monetary values of the human health benefits associated with air pollution removal. These assessments can be made for both individual trees and greenspace canopy cover. Furthermore, i-Tree Eco can forecast future benefits for some aspects such as carbon sequestration, air quality, and hydrology. The tool has been adapted for use in several countries, including Australia. However, some aspects, such as energy use reduction and air pollution impacts, rely on US-specific data and may not be applicable elsewhere. Notably, i-Tree Eco has additional limitations, including its failure to comprehensively incorporate various health benefits of trees and greenspace beyond air pollution removal. It also does not account for mental health, physical health (cycling and walking, obesity, cardiovascular diseases), heat-related diseases, and social interaction benefits. Additionally, certain direct economic benefits of trees and greenspace such as increased property value and tourism-related gains are not included in its estimations.

The Melbourne Green Factor Tool, created in 2019, was founded upon a thorough review of the existing literature concerning the structural and functional aspects of greenspace. Its primary function is the evaluation and scoring of private building development proposals in Melbourne, with a focus on health and environmental benefits arising from the incorporation of greenspace within these proposals. This tool has several strengths, including its accessibility as a web-based, publicly available resource. It encompasses a wide spectrum of environmental benefits, such as mitigating urban heat islands, providing habitats, managing runoff, and supporting local food supplies. Moreover, it recognizes various health benefits associated with greenspace, including sharing opportunities for recreation, enhancing sense of place, fostering social cohesion, enhancing aesthetics, and contributing to mental well-being. The development of the tool follows a collaborative codesign and codevelopment approach, bringing together experts from various disciplines, including policy makers, sustainable building and landscape practitioners, software designers, and researchers. Notably, the tool is integrated into the urban planning process of the City of Melbourne.

However, it is essential to acknowledge several limitations of Melbourne Green Factor Tool. One notable limitation is that it does not account for the economic values associated with the health and environmental benefits or the direct economic benefits of trees and greenspace. It also relies on evidence available only up to 2019, making it incapable of adapting to new and evolving data. Furthermore, it primarily concentrates on assessing benefits of aerial greenspace, making it unsuitable for evaluating individual trees. Additionally, it falls short in quantifying physical health benefits, such as increased physical activities, cardiovascular health, and the prevention of obesity. Additionally, the tool does not take into account air purification benefits from greenspace, which was initially proposed but later removed during the peer-review process. The authors’ rationale for this omission was based on the belief that air pollution is not a significant issue in countries like Australia. They also wanted to ensure that the tool does not imply that greenspace is meant to address air quality problems. Nonetheless, it is important to note that while Australia generally experiences low annual air pollution levels, during extreme episodes such as bushfires, some hotspot areas have recorded some of the world’s highest pollution levels [71]. Each year, air pollution is estimated to result in thousands of hospitalizations and premature deaths in Australia [72].

In summary, this scoping review has several important implications for urban planning decision-making processes. Firstly, this review underscores a substantial gap in current urban planning decision-making processes, indicating that despite the abundance of evidence and a supportive policy environment, the reviewed frameworks and tools do not adequately consider the health and environmental benefits offered by trees and greenspace. This is in line with previous studies that highlighted that ecosystem services are often overlooked within the context of urban planning decisions [37,38,49]. This oversight suggests a potential failure to harness the positive impacts of trees and greenspace on public health and the environment in urban planning endeavors [73], leading to urban environments that can lead to significant inequalities and health problems [74]. Secondly, this review emphasizes the scarcity of tools that incorporate the economic values associated with the health and environmental benefits of trees and greenspace. Consequently, under existing approaches, trees and greenspace are perceived as liabilities rather than recognized for their potential economic contributions [75]. In the absence of such comprehensive tools, key urban planning decision makers, including planners, transport planners, engineers, and asset managers, often resort to financial decision-making tools to shape project structures and values. This could lead to urban planning decisions that prioritize the removal of trees and greenspace from projects in favor of economic viability. The overall implication is a call for the development and integration of comprehensive decision-making tools that consider the holistic benefits of trees and greenspace in urban planning. This includes tools that not only acknowledge the health and environmental advantages but also incorporate their economic values. Addressing this gap is crucial for creating sustainable, resilient, and people-centric urban environments.

It is essential to recognize certain potential challenges that must be considered in the future development of decision support tools for urban planning. One primary challenge is the quantitative assessment of certain benefits associated with trees and greenspace, such as mental health benefits. This challenge stems from methodological complexities, primarily due to the involvement of numerous confounding factors [31]. Another challenge is that costs and benefits related to preserving and expanding green areas and trees are unevenly distributed among different groups and separated over time. For instance, in the case of new development projects, the expense of preserving existing trees is borne upfront by the developer or the buyer, while the benefits accrue to the local community residing in the neighborhood over many years, making preserving trees less attractive to the developer or buyer at the time. Similarly, the cost of removing a mature tree, though seemingly low, fails to account for the loss of multiple health and environmental benefits provided by these trees over time. For any future decision support frameworks, models, and tools in the context of urban planning, it is imperative to acknowledge and address these challenges to ensure effective adaptation and sustainable development.

Several recommendations for improving urban planning processes and decision making can be drawn from the findings of this study. Firstly, it is important to integrate health, environmental, and economic dimensions of tree and greenspace benefits into urban planning decision-making processes. This can be achieved through the development of comprehensive frameworks and tools that evaluate and quantify multiple benefits of trees and greenspace. This review identified two comprehensive tools—i-Tree Tools and the Melbourne Green Factor Tool—which could serve as valuable starting points for this endeavor. Secondly, urban planning authorities should prioritize improving the capacity and expertise of technical professionals to include health and environmental benefits in assessments of urban planning scenarios and options. Thirdly, urban planning authorities could implement mandatory requirements for urban developers to incorporate a comprehensive evaluation of the broader health and environmental benefits of trees and greenspace into their project design and implementation processes. Urban planners should develop urban design and transport planning guidelines that incorporate the latest evidence regarding health and environmental benefits of trees and greenspace. To address the uneven distribution of costs and benefits of trees and greenspace, urban planning authorities could implement policies that require developers to pay for part of cost of preserving existing trees or planting new trees in underserved communities.

Future research directions should include the development of more comprehensive frameworks and tools for evaluating the multifaceted benefits of trees and greenspace. Future research should prioritize developing methodologies for quantifying the mental health benefits of trees and greenspace. This includes addressing the methodological challenges associated with quantifying these benefits by conducting longitudinal studies.

The primary strength of this study lies in our utilization of a thorough and transparent search strategy, developed in collaboration with an experienced research librarian. However, it is essential to acknowledge the limitations inherent in a scoping review, which include a restricted number of databases and potential biases during the screening and selection of studies. This scoping review is also limited as it assesses the frameworks and tools within the included studies exclusively based on their incorporation of health, environmental, and economic benefits associated with trees and greenspace. While we gathered information on other critical criteria, such as the tools’ development process (e.g., the depth of evidence and the participatory nature of the approaches employed), adaptability to evolving evidence, accessibility, and digital format availability, we did not employ these criteria for scoring and ranking the frameworks and tools. Despite these limitations, our study offers an extensive overview of the current decision-making landscape, shedding light on the significant gaps within the existing frameworks and tools in terms of their comprehensiveness in addressing the health, environmental, and economic benefits of trees and greenspace.

## 5. Conclusions

In conclusion, our scoping review has shed light on the state of urban planning decision-making processes concerning the integration of health, environmental, and economic benefits associated with trees and greenspace. The findings underscore a significant gap in current practices, as none of the studies examined reported processes that adequately and comprehensively incorporated all three dimensions of these benefits. It is therefore no surprise that preserving trees or increasing the quality and quantity of greenspace, more often than not, become the second or third priority in any new or existing development as there is no framework, model, or tool that can comprehensively assist decision makers.

This review highlights a pressing need for the development and adoption of more comprehensive urban planning decision-making processes that fully integrate health, environmental, and economic benefits associated with trees and greenspace. To move forward, urban planners, policy makers, and researchers should work together to bridge these gaps by creating and implementing frameworks and tools that take into account the multifaceted benefits of urban trees and greenspace. Importantly, future research in this area should focus to address the critical gap in terms of incorporating the true economic and monetary values of health and environmental benefits of trees and greenspace, which is crucial for informed and holistic urban development decision making.

## Figures and Tables

**Table 1:** Comprehensiveness of the reviewed frameworks/models/tools in terms of incorporating trees and greenspace benefits.

References	Trees and Greenspace Benefits (Score out of 3)			Total Score (Out of 9)
	Health	Environmental	Economic	
I-tree [43]	It only considers human health impacts of pollution removal. Does not account for increased physical activity, improved mental well-being, or increased social cohesion. (1)	Comprehensive—considers carbon sequestration and storage, hydrology effects (avoided runoff, interception, transpiration), habitat suitability. (3)	Incorporates building energy effects and monetary values of the human health benefits associated with air pollution removal. (2)	7
Teng, Wu [44]	Recreation focused—does not account for reduced heat-related illness. (1)	Considers biodiversity and water-way health. Does not consider carbon sequestration, avoided runoff, energy savings, avoided emissions, or temperature regulation. (2)	Incorporates economic considerations, but the economic values of the health and environmental benefits are not quantified. (1)	4
Brom, Engemann [45]	Considers reduced heat-related illness. Does not account for increased physical activity, improved mental well-being, or increased social cohesion. (1)	Considers biodiversity, reduced pollution, reduced runoff, and temperature regulation. Does not account for carbon sequestration, energy savings, or avoided emissions. (2)	Incorporates economic considerations, acknowledges economic implications of climate change, but does not quantify the economic values of health or environmental benefits. (1)	4
Gohari and Ahmad [46]	Considers generic “health benefit”—no specifics. (1)	Considers reduced air and noise pollution, decreased urban heat island, runoff management, and biodiversity benefits. Does not account for carbon sequestration, habitat and food provision, or groundwater replenishment. (2)	Considers direct economic values such as increased property value but does not quantify the economic values of health or environmental benefits. (1)	4
Hosseinpour, Kazemi [47]	None. (0)	Considers biodiversity. Does not include temperature regulation, carbon sequestration, avoided runoff, avoided emissions, energy savings, or pollution removal. (1)	Incorporates economic considerations, but economic values of the environmental benefits not quantified, health benefits not present. (1)	2
Nestico, Passaro [48]	Measures based on usability and ability to effect quality of life. Does not specifically address increased physical activity, reduced heat-related illness, increased social cohesion, or increased mental well-being. (1)	Measures based on accessibility and size of greenspace. Does not account for carbon sequestration, avoided runoff, pollution removal, energy savings, avoided emissions, or temperature regulation. (1)	Incorporates economic considerations, but economic values of the health and environmental benefits not quantified. (1)	3
Cortinovis and Geneletti [49]	Considers temperature regulation but not specifically in relation to reduced heat-related illness. Does not account for increased physical activity, improved mental well-being, or increased social cohesion. (1)	Considers reduced pollution, temperature regulation, carbon sequestration, and avoided runoff. Does not account for energy savings or avoided emissions. (2)	Acknowledges economic implications of the environmental benefits but does not quantify the health or environmental benefits. (1)	4
Grafakos, Gianoli [50]	Considers generic “health benefit”—no specifics. (1)	Considers biodiversity, climate change, and generic water, land, and air benefits. Does not specifically consider reduced pollution, carbon sequestration, avoided runoff, energy savings, avoided emissions, or temperature regulation. (2)	Incorporates economic considerations, but the economic values of the health and environmental benefits are not quantified. (1)	4
Tapsuwan, Marcos-Martinez [51]	None. (0)	Comprehensive—considers carbon sequestration, avoided runoff, pollution removal, energy savings, avoided emissions, and temperature regulation. (3)	Acknowledges economic implications of the environmental benefits but does not quantify them. Health benefits not present. (1)	4
Bush [52]	Considers increased physical activity and social cohesion. Does not account for reduced heat-related illness or increased well-being/decreased mental health issues. (2)	Comprehensive—considers temperature regulation, reduced runoff, and reduced pollution. Does not explicitly account for carbon sequestration, energy savings, or avoided emissions. (3)	None. (0)	5
Keyvanfar, Shafaghat [53]	Focused on “Walkability”—does not account for other health benefits, e.g., reduced heat-related illness. (1)	None. (0)	None. (0)	1
Koehler, Latshaw [54]	Comprehensive—considers increased physical activity, reduced heat-related illness, increased social cohesion, and increased mental well-being. (3)	None. (0)	None. (0)	3
Nelli, Schehl [55]	None. (0)	Relevant only to habitat suitability of one species. (1)	None. (0)	1
Reinwald, Ring [56]	None. (0)	UHI-focused—does not account for avoided runoff, energy savings, or pollution removal. (1)	Acknowledges economic implications of the environmental benefits but does not quantify them. Health benefits not present. (1)	2
Turhan, Atalay [57]	None. (0)	UHI-focused—does not account for avoided runoff, energy savings, or pollution removal. (1)	Incorporates economic considerations, but economic values of the environmental benefits not quantified and health benefits not present. (1)	2
Babí Almenar, Petucco [58]	None. (0)	Comprehensive—considers carbon sequestration, avoided runoff, pollution removal, energy savings, avoided emissions, and temperature regulation. (3)	Acknowledges economic implications of the environmental benefits but does not quantify them. Health benefits not present. (1)	4
Ramyar [59]	Recreation-focused—does not account for reduced heat-related illness. (1)	Considers carbon sequestration, reduced pollution, temperature regulation, and reduced runoff. Does not account for avoided emissions or energy savings. (2)	Incorporates economic considerations, but the economic values of the health and environmental benefits are not quantified. (1)	4
Ren, Lau [60]	None. (0)	UHI-focused—does not account for avoided runoff, energy savings, or pollution removal. (1)	None. (0)	1
Pena Acosta, Vahdatikhaki [61]	None. (0)	UHI focused—does not account for avoided runoff, energy savings, or pollution removal. (1)	None. (0)	1
Lopucki and Kiersztyn [62]	None. (0)	Relevant only to habitat suitability of small mammals. (1)	None. (0)	1
Tiwary, Williams [63]	None. (0)	Comprehensive—considers carbon sequestration, avoided runoff, pollution removal, energy savings, avoided emissions, and temperature regulation. (3)	None. (0)	3
Badach and Raszeja [64]	Accounts for visual indicators which may have health benefits present but does not explicitly mention their connection to health. Does not account for increased physical activity, reduced heat-related illness, or increased social cohesion. (1)	Considers temperature regulation and pollution removal. Does not account for carbon sequestration, avoided runoff, energy savings, or avoided emissions. (2)	None. (0)	3
Fernández and Wu [65]	Considers heat-related illness. Does not account for increased physical activity, increased mental well-being, or increased social cohesion. (1)	Considers temperature regulation and reduced pollution. Does not account for carbon sequestration, avoided emissions, energy savings, or avoided runoff. (2)	Incorporates economic considerations, but the economic values of the health and environmental benefits are not quantified. (1)	4
Vallecillo, Polce [66]	Considers generic “health benefit”—no specifics. (1)	Considers biodiversity and reduced pollution. Does not consider carbon sequestration, reduced runoff, avoided emissions, energy savings, or temperature regulation. (2)	Incorporates economic considerations, acknowledges that there is economic value to health and environmental benefits but does not quantify them. (1)	4
Bellamy, van der Jagt [67]	Measures physical and mental health through hospital stays, mortality, and rates of anxiety, depression, and psychosis. Does not specifically account for social cohesion. (2)	Focuses on benefits for pollinators. Does not account for carbon sequestration, avoided runoff, pollution removal, energy savings, avoided emissions, or temperature regulation. (1)	None. (0)	3
Aldea, Luca [68]	UHI focused—does not account for other health benefits. (1)	UHI-focused—does not account for avoided runoff, energy savings, or pollution removal. (1)	None. (0)	2
Fu, Hopton [69]	Measures based on “increased recreation area”. Does not explicitly account for increased physical activity, improved mental well-being, or increased social cohesion. Does not account for reduced heat-related illness. (1)	Considers reduced runoff and reduced pollution. Does not account for temperature regulation, avoided emissions, energy savings, or carbon sequestration. (2)	Incorporates economic considerations, but the economic values of the health and environmental benefits are not quantified. (1)	4
Srdjevic, Lakicevic [70]	Considers increased physical activity and social cohesion. Does not account for reduced heat-related illness or increased well-being/decreased mental health issues. (2)	Considers temperature regulation and reduced pollution. Does not account for carbon sequestration, avoided runoff, energy savings, or avoided emissions. (2)	None. (0)	4

Key: 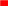 does not include any benefit from this column; 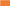 includes some benefits from this column but is not comprehensive; 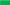 tool comprehensively includes all relevant benefits from this column.

## Data Availability

Not applicable.

## Appendix A. Search Strategy

**Table A1:** Master Logic Grid.

Greenspace	Decision-Making Processes	Urban Planning
Green space* Trees Green belt* Parkland* Urban Green* Greenif* Greenspace Green infrastructure Green-space*	Decision procedure* Decision logic Decision mak* Decision matrix Decision tool* Decision framework* Planning tool* Planning framework*	Town plan* City plan* Urban development Environmental design Local plan*

**Table A2:** PubMed Logic Grid.

Green Space (s)	Decision-Making Processes	Urban Planning
Green space*[tiab] OR Trees[tiab] OR Green belt*[tiab] OR Parkland*[tiab] OR Urban green*[tiab] OR Greenif*[tiab] OR Greenspace[tiab] OR Green infrastructure[tiab] OR Green-space* OR “Parks, Recreational”[mh]	Decision procedure*[tiab] OR Decision logic[tiab] OR Decision mak*[tiab] OR Decision matrix[tiab] OR Decision tool*[tiab] OR Decision framework*[tiab] OR Planning tool*[tiab] OR Planning framework*[tiab] OR “Decision Making”[mh] Planning framework*	Town plan*[tiab] OR City plan*[tiab] OR Urban development[tiab] OR Environmental design[tiab] OR Local plan[tiab] OR “City Planning”[mh] OR “Environment Design”[mh]

**Table A3:** Scopus Logic Grid.

Greenspace	Decision-Making Processes	Urban Planning
TITLE-ABS-KEY(“Green space*” OR Trees OR “Green belt*” OR Parkland* OR “Urban Green*” OR Greenif* OR Greenspace OR “Green Infrastructure” OR Green-space)	TITLE-ABS-KEY(“Decision mak*” OR “Decision procedure*” OR “Decision logic” OR “Decision matrix” OR “Decision tool*” OR “Decision framework*” OR Planning tool*” OR “Planning framework*)	TITLE-ABS-KEY(“Urban planning” OR “Town planning” OR “City planning” OR “Urban development” OR “Environmental design” OR “Local plan*”)

**Table A4:** Public Health Database Logic Grid.

Greenspace	Decision-Making Processes	Urban Planning
“Green space*” OR Trees OR “Green belt*” OR Parkland* OR “Urban Green*” OR Greenif* OR Greenspace OR “Green Infrastructure OR Green-space”	“Decision mak*” OR “Decision procedure*” OR “Decision logic” OR “Decision matrix” OR “Decision tool*” OR “Decision framework*” OR “Planning tool*” OR “Planning framework*”	“Urban planning” OR “Town planning” OR “City planning” OR “Urban development” OR “Environmental design” OR “Local plan*”

**Table A5:** Web of Science Logic Grid.

Greenspace	Decision-Making Processes	Urban Planning
“Green space*” OR Trees OR “Green belt*” OR Parkland* OR “Urban Green*” OR Greenif* OR Greenspace OR “Green Infrastructure OR Green-space”	“Decision mak*” OR “Decision procedure*” OR “Decision logic” OR “Decision matrix” OR “Decision tool*” OR “Decision framework*” OR “Planning tool*” OR “Planning framework*”	“Urban planning” OR “Town planning” OR “City planning” OR “Urban development” OR “Environmental design” OR “Local plan*”

**Table A6:** Google Advanced Search Logic Grid.

Greenspace	Decision-Making Processes	Urban Planning
(“Green space*” OR Trees OR “Green belt*” OR Parkland* OR “Urban Green*” OR Greenif* OR Greenspace OR “Green Infrastructure”)	(“Decision procedure*” OR “Decision logic” OR “Decision matrix” OR “Decision mak*”)	(“Urban plan*” OR “Town plan*” OR “City plan*” OR “Urban development” OR “Environmental design” OR “Local plan*”)

## Appendix B. Summary Results of Publications Included in the Scoping Review

**Table A7:** Extracted Descriptive Data for Evaluated Frameworks, Models, and Tools in the Scoping Review.

References	Year	Country	Methodology	Health Benefits (y/n)	Environmental Benefits (y/n)	Economic Benefits (y/n)	Strengths	Limitations
[43]	2006	USA	i-tree is a peer-reviewed software suite of tools which provides a range of urban forestry analyses and benefit assessment tools. Tools are based on primary data.	Yes	Yes	Yes	Free, user-friendly, available to urban planners as well as the general public.	Some estimates are based on US-specific data.
[44]	2011	China	Least-cost path model (LCP) uses a raster-based algorithm which develops the least costly way to create multifunctional greenway networks. Remote sensing was used to collect primary data.	Yes	Yes	Yes	Priorities can be interchanged or weighted differently depending on the study setting.	Focuses on construction costs only, does not quantify the health and environmental costs/benefits. Land surface temperature maps may have reflected unwanted factors; they should be optimized before being used again in the future.
[45]	2023	South Africa	GIS-based decision support tool that integrates urban planning objectives for climate resilience and biodiversity. Tool was developed using the following steps: identify multifunctional benefits together with stakeholders; select maps to represent each benefit; engage stakeholders to determine relative importance of each benefit; assembly and quality control; stakeholder workshop to discuss applications, opportunities, and limitations of tool.	Yes	Yes	Yes	Considers local stakeholder priorities. Treats ecological and human health as interconnected concepts (e.g., considers the impact of climate on human health). Also acknowledges the economic implications of climate and accessibility.	Does not include ranking of greenspace based on user preference. Only considers spatial access, not access based on social and cultural relations.
[46]	2023	Iran	Framework for creating an MCDM tool used to determine the social, economic, and environmental costs and benefits of green roofs. Best worth method (BWM) was used to prioritize indicators. Surveys were completed by experts in the field to determine weighting for indicators.	Yes	Yes	No	Incorporates social, environmental, and economic factors and assigns weightings to them. Separates different types of buildings—i.e., governmental buildings and residential buildings.	Does not quantify the economic/monetary values of improved/reduced human and environmental health. Limited to green roofs rather than multiple types of green infrastructure and greenspace. Is a framework for developing a tool rather than a tool itself.
[47]	2022	Iran	Value engineering (VE), multicriteria decision-making (MCDM), and risk management (RM) methods were integrated to create a framework. This framework is used to determine the profitability of urban agriculture in comparison with conventional urban park design.	No	Yes	Yes	Combination of VE, MCDM, and RM methods has created a comprehensive framework.	Discusses economic implications of greenspace but does not quantify the health and environmental costs/benefits. Human health implications of greenspace are not considered. Value weightings were chosen by experts but could vary, depending on which experts were asked, as well as the monetary value differences between countries.
[48]	2022	Italy	Tool combining four decision-making methods (AHP, ELECTRE, TOPSIS, and VIKOR) to establish the best localization of greenspace. All four methods utilize qualitative and quantitative criteria and require weightings to be assigned to these criteria. Estimation of weights conducted by a diverse panel of experts who undertook pairwise comparisons of the evaluation criteria.	Yes	Yes	Yes	Indicators are straightforward and easy to estimate. Adaptable from one study setting to another as it is largely based on secondary data.	Investment costs and property are considered yet does not quantify the health and environmental costs/benefits. All four methods are MCDM methods—using a wider range of methods could have allowed for better comparison.
[49]	2019	Italy	Conceptual framework which helps urban planners to make decisions around greenspace based on the provision of urban regulating ecosystem services. The framework was built upon two existing models—the cascade conceptual model and the supply–demand approach for ecosystem mapping.	Yes	Yes	Yes	Quantifies costs/benefits of environmental implications of ecosystem services. Framework highlights to urban planners how their decisions will either enhance or reduce ecosystem service benefits. Systematizes available scientific knowledge and makes it available to urban planners.	Does not quantify costs/benefits of health implications of ecosystem services. The complexity of ecosystem services had to be simplified for the purposes of the framework, meaning some of the nuance was lost.
[50]	2016	The Netherlands	Sustainability and resilience benefits assessment (SRBA) identifies the sustainability benefits associated with a development. A systems-thinking approach was used to develop a matrix which demonstrates the type of sustainability and resilience benefits which can be expected from a project.	Yes	Yes	Yes	Integrates top-down and bottom-up approaches. Cities able to decide which sustainability indicators are most relevant to them.	Assesses growth, revenue, and balance of payment but does not quantify the health and environmental costs/benefits. Authors recognize that increasing sustainability in one area may inevitably decrease it in another, meaning that the tool does have some tradeoffs.
[51]	2021	Australia	The System of Experimental Ecosystem Accounting (SEEA) framework assigns monetary values to environmental assets in order to determine their worth. Estimates of monetary value were generated using i-tree, a literature review, and a series of nonmarket valuation estimations and benefit transfer estimations.	No	Yes	Yes	Tool interprets the “cost” of a natural asset in two ways; the physical value of the asset and the value of the ecosystem services it provides; thus quantifies the costs/benefits of environmental impacts of greenspace. Article highlights where further research/data are required.	Human health implications of greenspace are not considered. Some benefits were not able to be measured due to inadequate data.
[52]	2021	Australia	Melbourne Green Factor Tool assesses the benefit of a greenspace for environmental and human health. The web-based tool calculates a green factor score for a site based on weighted indicators. A literature review formed the evidence base for the tool.	Yes	Yes	No	Open access and user-friendly. Developed tool after comparative analysis of the strengths and limitations of similar tools.	Economic implications of greenspace are not considered. Chose not to include air quality as a function.
[53]	2018	Malysia	The WIND tool uses a comprehensive list of variables to evaluate an area’s walkability based on its environmental and physical attributes. The “most in use” philosophical approach was used to create the tool along with mind-mapping and decision-tree methods to collect primary data from pedestrians.	Yes	No	No	Provides scored index to benchmark the walkability of each urban neighborhood, assisting urban planners in prioritization.	Environmental and economic factors not considered. Potential for biodiverse greenspace in walkable areas has been missed—study only focuses on walkability.
[54]	2018	USA	A framework which guides urban planners to make decisions which will improve community health. The framework presents a “current state” and a “desired state” and suggests a tool kit which can take a community from its current state to its desired state. These tools include a health impact assessment, public health tracking, EPA EnviroAtlas and C-FERST, and cumulative risk assessment.	Yes	No	No	Framework shows a clear path from “current state” to “desired state” and includes steps on how to achieve this.	Economic burden of environment-related disease is acknowledged yet not included in the framework. Environmental factors not considered.
[55]	2022	Scotland	The habitat suitability model was used to assess natural urban spaces for their ability accommodate water voles. This tool can be used to determine the implications of future developments on the ecosystem. Model was created using a resource selection probability function (RSPF) following a use versus availability design.	No	Yes	No	Available as a user-friendly web application. Potential for future development into a multispecies tool.	Health and economic implications of greenspace are not considered. Currently only focuses on one species—not a versatile tool.
[56]	2019	Austria	Combination of four different tools (GFF, GREENPASS, MUKLIMO_3, and Cosmo-CLM) into a tool set which assesses the impact of UHI.	No	Yes	Yes	Combination of four tools makes it possible to assess dynamic scenarios (e.g., the tool could provide the best building type in combination with the best greenspace type).	Human health implications of greenspace are not considered. Limited assessment of economic aspects of greenspace. Tools in the set must still be operated individually—their integration into a single, comprehensive tool requires further research.
[57]	2023	Turkey	UHI mitigation framework which integrates a microclimatic simulation, a dynamic energy performance simulation, and a multicriteria decision-making (MCDM) tool.	No	Yes	Yes	Tools other than the MCDM can be used interchangeably in the framework. Integrated framework allows for multidimensional approach.	Discusses economic implications of energy use but does not quantify environmental impacts of greenspace. Human health implications of greenspace are not considered. Air temperature is the only factor assessed in the mitigation of UHI, though meteorologists suggest that wind speed, radiant temperature, and humidity are all relevant. The researchers also acknowledged that the study was conducted too close to buildings, thus potentially skewing the results.
[58]	2023	Spain	Online decision support tool which calculates the positive and negative environmental impacts, externalities, and financial values of planned urban forests. The software uses an underpinning modeling framework that combines system dynamics, urban ecology, and life cycle thinking methods.	No	Yes	Yes	Tool is able to be adapted to calculate for various types of greenspace/green infrastructure. Environmental implications of greenspace are quantified.	Human health implications of greenspace are not considered. Still work to be performed to ensure the tool is user-friendly.
[59]	2019	Iran	ArcGIS spatial tool is used to create ecosystem service maps of urban parks, urban gardens, urban farmland, and vacant lots. The tool uses a multicriteria approach to assess the supply and demand of ecosystem services by geographical area.	Yes	Yes	Yes	Integrates social and ecological ecosystem services provided by greenspace. Priority map shows which neighborhoods need these ecosystem services the most.	Prioritizes greenspace based on socio-economic need; however, does not quantify the health and environmental costs/benefits. Iran has limited ecosystems services data, so this approach may look different in different study settings.
[60]	2013	Taiwan	Urban climatic map (UCmap) provides a visual and spatial platform for the geographical information system (GIS). Together, these tools map problem areas/sensitive areas where population density and UHI intensity are of issue and where there is potential for greenspace.	No	Yes	No	Easy to use and has a flexible structure. Is an interdisciplinary tool—and can be used by urban planners and policy makers alike.	Mentions effects of UHI on human health but does not incorporate this in the tool. Does not incorporate economic dimensions.
[61]	2021	Canada	The tool classifies streets based on their UHI from low to high and provides guidance on where additional vegetation would assist in lowering UHI. A data-driven modeling approach was used to develop the tool.	No	Yes	No	Publicly available data can be used to operate the decision matrix and categorize streets based on their UHI impact. The five levels of classification are straightforward and easy to interpret.	Health and economic implications of greenspace are not considered. Had air temperature been used to determine UHI rather than SUHI, the results may have been different—this should be investigated in future research.
[62]	2015	Poland	Hellwig’s method was applied to create a mathematical algorithm tool which identifies the ecological significance of urban greenspace and their groupings. Data were derived from the literature as well as primary data collected specifically for the study.	No	Yes	No	Would improve transparency in urban planning, could potentially be used to mitigate conflict in planning, provides the opportunity for standardized rankings to definitively and transparently present options.	Health and economic implications of greenspace are not considered. Hellwig’s method treats multiple similar greenspaces as redundant and would therefore advise to remove them as they do not add *different* biodiversity, even though they add *more* biodiversity. The method also does not account for areas acting as ecological corridors or connectivity between greenspace networks. Particular cases therefore require further evaluation by urban ecologists.
[63]	2016	EU	The performance index framework was created to evaluate the effects of different streetscapes on environmental health. Conceptual framework was based on a literature review. Primary field survey data and data extracted from the literature were then used to test the framework.	No	Yes	No	Applicable for evaluating existing streetscapes and planning new ones. The index is a repeatable metric, allowing for comparative evaluations of greenspace.	Health and economic implications of greenspace are not considered. Not a comprehensive framework yet—still lacks data on storm water runoff. Assumes one plant species per streetscape rather than allowing for variety. Also does not account for seasonality of streetscapes—e.g., certain species may have a higher PI at certain times of year.
[64]	2019	EU and UK	A landscape and green infrastructure framework with multiple indicators sorted into three groups—ecological, visual, and structural. These indicators were identified based on a literature review, and their contextual applicability was tested through document review and case studies.	Yes	Yes	No	The framework has long-term uses. Considered visual indicators which may indirectly influence healthy activities.	Does not consider economic indicators. Health indicators are neither comprehensive nor explicit.
[65]	2018	Chile	Environmental improvement priority index (EIPI) identifies key areas to target with environmental inequity mitigation measures. EIPI is constructed through the integration of two spatial indicators: an environmental stress indicator (ESI) and a social relevance indicator (SRI). Conceptual framework was based on a literature review. Framework was tested in a case study using secondary data.	Yes	Yes	Yes	Prioritizes greenspace in low socio-economic areas as disadvantaged people are more likely to experience the negative effects of environmental inequity. “Holistic” approach accounts for the social and economic aspects of environmental inequality. Can be applied at different spatial and administrative levels.	Does not quantify the health and environmental costs/benefits. Relies on prior research having been performed to identify environmental inequity problems in the area. Participatory process with stakeholders for further development of tool should be undertaken during future research.
[66]	2018	EU	Prioritization framework which determines the allocation of green infrastructure which best supports human and ecosystem health. Spatial conservation prioritization (SCP) methods are used to identify key socio-economic areas and areas in poor ecosystem condition. Literature-based scenario definition and secondary data-based modeling were used.	Yes	Yes	Yes	Acknowledges that human and ecosystem health are inextricably linked, as well as considering the cost effectiveness of green infrastructure.	Focuses on cost effectiveness but acknowledges that there are economic benefits to the environment and human health. Cost effectiveness of removing invasive species requires more information as it is currently based on assumptions.
[67]	2017	Scotland	The habitat suitability model (HSM) is used to determine what types of green infrastructure would be most beneficial for human and pollinator health, as well as where they should be put. Predictive maps highlight areas where both human and pollinator health could be improved, creating a “win win” scenario. Stakeholder engagement and secondary data were used to develop the tool.	Yes	Yes	No	Stakeholder engagement was undertaken. Framework can be adapted for different study settings and different pollinators.	Economic implications of greenspace are not considered.
[68]	2018	Romania	WebGIS mapping portal for urban climate determines the impact of UHI on human health and comfort. To achieve this, remote sensing, geographical information systems, crowdsourcing, and microclimatic modeling were utilized.	Yes	Yes	No	Is accessible enough to be used by the public as well as urban planners. Transparent.	Economic implications of greenspace are not considered. E-participation is encouraged, which can be a good way of engaging the public but can also invite unhelpful or irrelevant information.
[69]	2021	USA	The GIUR-PSS framework assesses scenarios for their environmental, social, economic, and cultural dimensions of resilience. This tool not only assesses these three aspects of green infrastructure but combines qualitative and quantitative indicators using the fuzzy comprehensive evaluation (FCE) method. The assessment process is embedded in a planning support system (PSS) which combines geospatial data, methods, and technologies to assist with decision making in planning. Literature-based scenario generation and indicator set was coupled with either expert surveys or modeling using primary and secondary data.	Yes	Yes	Yes	Multidimensional approach assesses for social, economic, environmental, and cultural dimensions of resilience. Can also incorporate both qualitative and quantitative factors in a scenario assessment.	Acknowledges the financial costs/benefits of greenspace (e.g., maintenance) but does not quantify the health and environmental costs/benefits. Scenarios can only assess one type of green infrastructure in one study area—they cannot assess one study area with multiple types of green infrastructure.
[70]	2013	Serbia	The analytic hierarchy process (AHP) model was used in conjunction with a group decision-making framework and the consensus convergence model (CCM) in order to make decisions with regard to urban planning. Preexisting framework was customized and then tested by a team of experts.	Yes	Yes	No	Easy-to-understand and -operate approach.	Economic implications of greenspace are not considered. Requires all decision makers in the group to reach a consensus.
